# Optimization of *Helicobacter pylori* Biofilm Formation in In Vitro Conditions Mimicking Stomach

**DOI:** 10.3390/ijms25189839

**Published:** 2024-09-11

**Authors:** Paweł Krzyżek, Paweł Migdał, Barbara Krzyżanowska, Anna Duda-Madej

**Affiliations:** 1 Department of Microbiology, Faculty of Medicine, Wroclaw Medical University, 50-368 Wroclaw, Poland; barbara.krzyzanowska@umw.edu.pl (B.K.); anna.duda-madej@umw.edu.pl (A.D.-M.); 2 Department of Bees Breeding, Institute of Animal Husbandry, Wroclaw University of Environmental and Life Sciences, 51-630 Wroclaw, Poland; pawel.migdal@upwr.edu.pl

**Keywords:** *Helicobacter pylori*, *Helicobacter pylori* biofilm, KATO III, host-mimicking conditions, host-mimicking fluids, culture conditions

## Abstract

*Helicobacter pylori* is one of the most common bacterial pathogens worldwide and the main etiological agent of numerous gastric diseases. The frequency of multidrug resistance of *H. pylori* is growing and the leading factor related to this phenomenon is its ability to form biofilm. Therefore, the establishment of a proper model to study this structure is of critical need. In response to this, the aim of this original article is to validate conditions of the optimal biofilm development of *H. pylori* in monoculture and co-culture with a gastric cell line in media simulating human fluids. Using a set of culture-based and microscopic techniques, we proved that simulated transcellular fluid and simulated gastric fluid, when applied in appropriate concentrations, stimulate autoaggregation and biofilm formation of *H. pylori*. Additionally, using a co-culture system on semi-permeable membranes in media imitating the stomach environment, we were able to obtain a monolayer of a gastric cell line with *H. pylori* biofilm on its surface. We believe that the current model for *H. pylori* biofilm formation in monoculture and co-culture with gastric cells in media containing host-mimicking fluids will constitute a platform for the intensification of research on *H. pylori* biofilms in in vitro conditions that simulate the human body.

## 1. Introduction

*Helicobacter pylori* is one of the most common bacterial pathogens worldwide and the main etiological factor in chronic progressive gastritis, which over time determines the development of numerous gastroenterological diseases [1]. *H. pylori* infection is often acquired in childhood and progresses to a life-long state if not successfully treated. Amoxicillin, metronidazole, clarithromycin, levofloxacin, and tetracycline are the most commonly used antibiotics, although in recent years bismuth salts have also been gaining importance and are applied during quadruple therapy [2].

The success of treatment directed against *H. pylori* is highly dependent on the level of antibiotic resistance [3,4]. Unfortunately, together with a global increase in antibiotic resistance incidence, achieving therapeutic efficacy becomes a real challenge and critical issue [5,6]. Mechanisms negatively influencing the possibility of *H. pylori* eradication include patient adherence (availability of drugs, cost of the treatment, side effects), host-related factors (genetic determinants, immune status), and factors associated with a bacterium (virulence, resistance profile, density in the stomach) [3,7]. For *H. pylori*, it is indicated that resistance to single antibiotics is associated with point mutations in drug target sites, while multidrug resistance is strongly related to biofilm formation [6,8].

A biofilm is defined as multicellular communities of microorganisms surrounded by an extracellular matrix [9,10]. The extracellular matrix is a mixture of various biomacromolecules, including polysaccharides, proteins, lipids, and nucleic acids. Its presence determines a number of phenotypic features of biofilm cells that are beneficial to microorganisms, i.e., increased aggregation, intensified adhesion to biotic and abiotic surfaces, and decreased sensitivity to antibiotics or activity of the immune system. The state of knowledge regarding biofilm production by *H. pylori*, although still quite basic in relation to many other bacterial species, has expanded significantly in the last decade [11,12,13,14,15]. Disturbingly, recent reports indicate a link between antibiotic resistance and/or tolerance and biofilm formation of *H. pylori* [16,17,18,19,20,21]. The relevance of the above-mentioned phenomenon is well reflected by the promising data of Cammarota et al. [22], who proved in a randomized control trail that N-acetylcysteine-dependent disruption of *H. pylori* biofilm before culture-guided therapy was significantly associated with a better treatment efficacy of patients. Therefore, the intensification of research on biofilm produced by *H. pylori* becomes undoubtedly even more important.

Systems commonly applied to develop and analyze biofilms are characterized by high throughput capabilities, but suffer from a lack of proper simulation of in vivo conditions [23,24]. Simplified research models do not have the appropriate factors related to the host (substrate, medium flow, or biochemical conditions), and this certainly limits the possibility of directly translating the results obtained in the laboratory into a real-life scenario [24]. The use of animal models in biofilm research, although allowing for a relatively reliable representation of clinical conditions, is at the same time burdened with high costs and a number of bioethical issues [25]. In response to this, the use of host-mimicking fluids as culture media is becoming increasingly popular in studies focused on the determination of the physiology or drug susceptibility of microorganisms [26,27]. This approach facilitates access to this type of research procedure for less-specialized or funded laboratories, while allowing them to conduct the replication of in vivo conditions. To our knowledge, the only research on *H. pylori* that has used simulated human fluids in the cultivation of this microorganism consists of several studies testing new therapeutics [28,29,30] and a single study verifying the involvement of urease in the motility of this bacterium in mucins [31]. The use of culture media imitating human fluids in research on *H. pylori* biofilm development has not yet been applied.

Therefore, the aim of this original article was to validate the conditions of optimal biofilm development of *H. pylori* in monoculture and co-culture with a gastric cell line in culture media simulating human fluids.

## 2. Results and Discussion

### 2.1. Selection of H. pylori Strain

For this research, we decided to select a clinical multidrug-resistant *H. pylori* 2CML, which in our previous studies had demonstrated the most intensive biofilm production among all tested strains [16]. As part of our previous studies [16,17], we also managed to demonstrate that its set of phenotypic features—rapid adaptation to changing environmental conditions expressed in dynamic autoaggregation and biofilm formation—make it an ideal strain to test the optimal culture conditions of our current research model.

### 2.2. Effect of Simulated Transcellular Fluid (STF) on the Physiology of H. pylori

**Figure 1:**
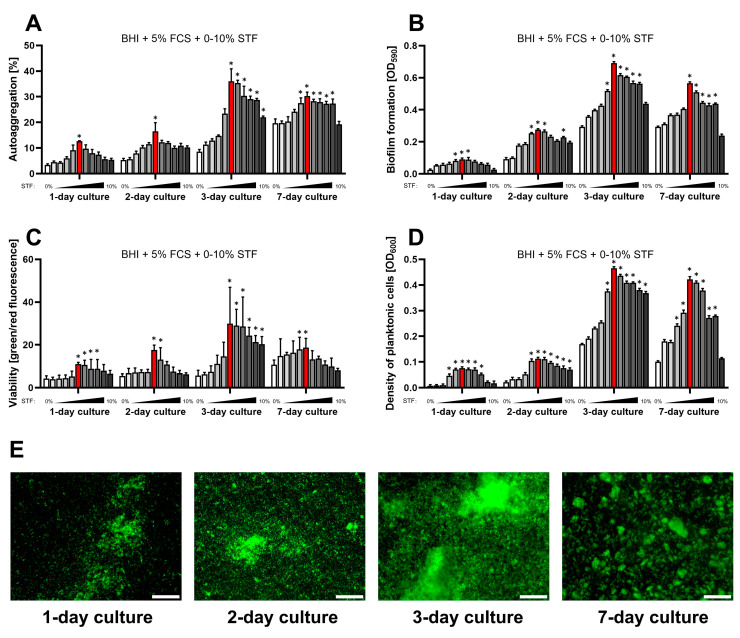
Assessment of the impact of a one-week culture on the physiological parameters of *H. pylori* 2CML in BHI + 5% FCS and the concentration gradient of STF (0–10%, with 1% intervals). An increasing concentration of STF is marked by the intensified gray of bars. (**A**) Autoaggregation measured microscopically by estimating the degree of the observation field coverage, *n* = 3. (**B**) Biofilm formation determined by a crystal violet staining method and spectrophotometric measurements, *n* = 9. (**C**) Viability of bacterial cells determined by fluorescent staining with a LIVE/DEAD kit and a ratio of green/red fluorescence, *n* = 3. (**D**) Density of planktonic cells assessed by spectrophotometry, *n* = 9. (**E**) A set of images showing the growth of *H. pylori* 2CML under the most optimal conditions of the current model—BHI + 5% FCS + 5% STF (marked with red bars). Scale bars, 20 µm. * indicates statistical difference (*p* < 0.05) with the control—0%.

Transcellular fluids constitute a part of extracellular fluids and are found within close proximity to epithelial cells, including the gastrointestinal tract [32]. They act mostly as a lubricant agent and help in electrolyte exchange. As the gastric epithelium is the natural place where *H. pylori* resides in the human body [33,34], we wanted to investigate the impact of transcellular fluid on this bacterium. In line with this, in the first stage of our model, we assessed whether the presence of a concentration gradient of STF (0–10%, in 1% intervals) in the classically used culture medium (Brain Heart Infusion broth (BHI) + 5% Fetal Calf Serum (FCS)) affects several physiological parameters of *H. pylori*, such as the optical density of planktonic forms, viability (measured with the LIVE/DEAD kit), autoaggregation degree, and the amount of biofilm formed (Figure 1).

We observed that concentrations between 1% and 5% of STF gradually stimulated all the biological parameters tested. Interestingly, this effect was maximized at 5% STF, after which further increasing of the concentration led to a reduction in all these indicators (Figure 1). We suspect that this phenomenon is the result of the nutritional enrichment of BHI with 5% FCS, and the addition of too-high concentrations of other components to this medium may cause bacterial cell death, e.g., osmotic lysis [35]. Regardless, the above observations indicate that the culture of *H. pylori* in the presence of STF (at an experimentally selected concentration) has an inducing effect on both the proliferation of planktonic cells and the amount of biofilm produced.

Another aspect worth noting is that 3-day culture under the above-mentioned conditions was optimal to maximize the development of *H. pylori* biofilm (Figure 1). A longer incubation period of 7 days contributed to the reduction in the biofilm amount and the transformation of its structure from single large-scale aggregates into smaller more numerous microaggregates. We suspect that this process of biofilm architecture rearrangement is related to the production of autoinducer 2 (AI-2) by *H. pylori*. AI-2 constitutes quorum sensing molecules, which are synthesized as the density of bacterial cells increases [36]. Interestingly, high concentrations of AI-2 are perceived by *H. pylori* as a chemorepellent signal, inducing cell dispersion from mature biofilm [37,38]. In the research of Sweeney et al., who used computer modeling to assess the impact of AI-2 on the structure of *H. pylori* biofilm, it was observed that the accumulation of these compounds was accompanied by the transformation of large biofilm aggregates into many smaller ones [39]. Therefore, we suspect that the perception of AI-2 by *H. pylori* might be associated with rearrangements of the biofilm structure in our experiments; however, a careful set of studies needs to be performed to confirm this hypothesis.

**Figure 2:**
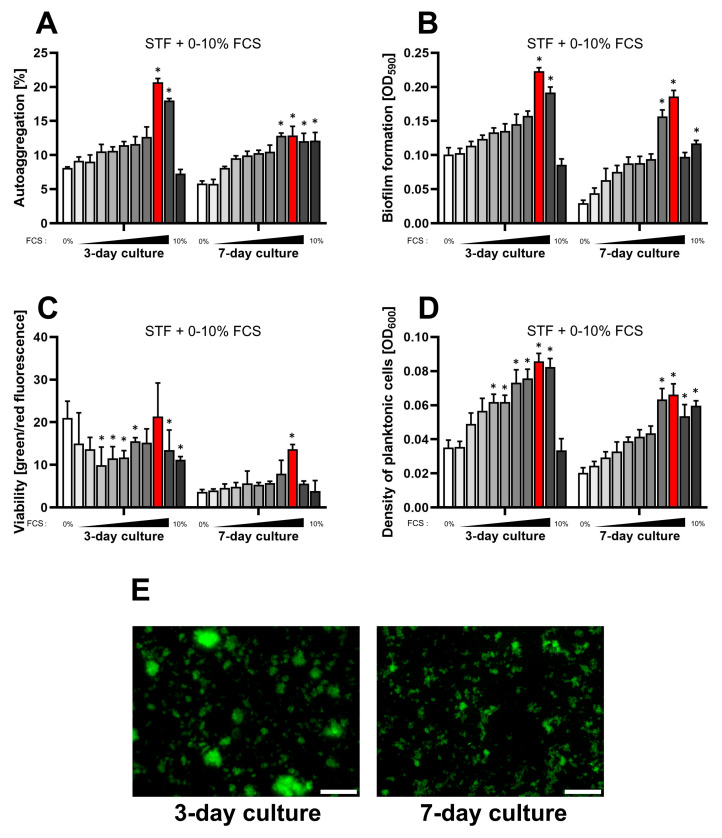
Assessment of the impact of a one-week culture on the physiological parameters of *H. pylori* 2CML in STF and the concentration gradient of FCS (0–10%, with 1% intervals). An increasing concentration of FCS is marked by the intensified gray of bars. (**A**) Autoaggregation measured microscopically by estimating the degree of the observation field coverage, *n* = 3. (**B**) Biofilm formation determined by a crystal violet staining method and spectrophotometric measurements, *n* = 9. (**C**) Viability of bacterial cells determined by fluorescent staining with a LIVE/DEAD kit and the ratio of green/red fluorescence, *n* = 3. (**D**) Density of planktonic cells assessed by spectrophotometry, *n* = 9. (**E**) A set of images showing growth of *H. pylori* 2CML under the most optimal conditions of the current model—STF + 8% FCS (marked with red bars). Scale bars, 20 µm. * indicates statistical difference (*p* < 0.05) with the control—0%. Because of the low values for all the physiological parameters during 1- and 2-day cultures, these data were not recorded.

In the second stage of this series of experiments, we decided to assess the physiology of *H. pylori* growing in STF as the primary culture medium. To increase bacterial multiplication, we tested whether the presence of FCS, and which concentration, is optimal under these culture conditions (Figure 2). In the case of three parameters (optical density of planktonic cells, autoaggregation degree, and biofilm amount), we noticed a linear correlation between the increase in the applied FCS concentration and the intensification of these biological processes. We detected the saturation of the effect at a concentration of 8% FCS, after which a steep decline in the above processes was noticeable. We suspect that, as in our previous experiments, a too high concentration of nutrients in the culture medium may lead to osmotic cell death. Again, we noticed that a 3-day culture had a more beneficial effect on the biological parameters assessed than a 7-day incubation period.

To sum up, we managed to confirm the validity of the use of STF as a stimulator of autoaggregation and biofilm formation of *H. pylori*, both in the standard BHI + FCS medium (Figure 1) and as a primary culture medium when supplemented with sufficient concentrations of FCS (Figure 2).

### 2.3. Effect of Simulated Gastric Fluid (SGF) on the Physiology of H. pylori

**Figure 3:**
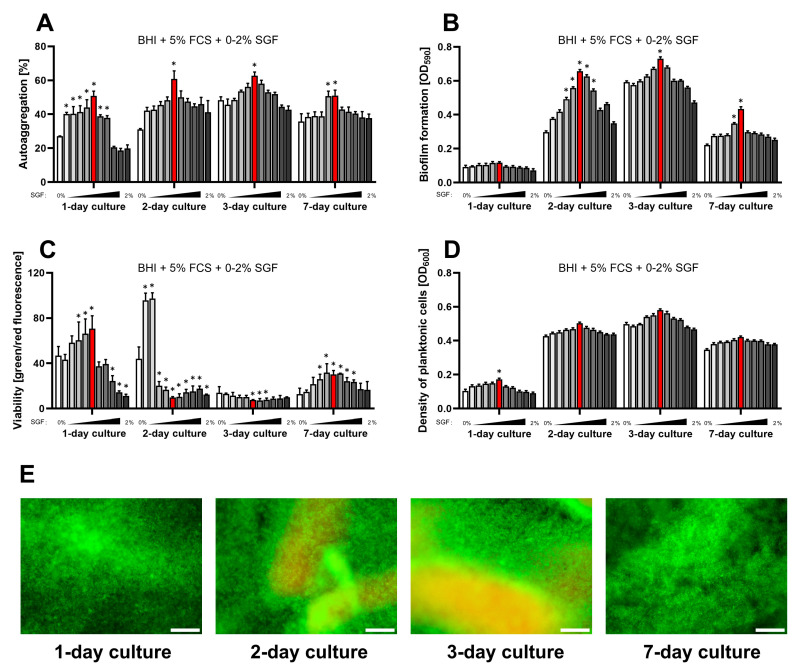
Assessment of the impact of a one-week culture on the physiological parameters of *H. pylori* 2CML in BHI + 5% FCS and the concentration gradient of SGF (0–2%, with 0.2% intervals). An increasing concentration of SGF is marked by the intensified gray of bars. (**A**) Autoaggregation measured microscopically by estimating the degree of the observation field coverage, *n* = 3. (**B**) Biofilm formation determined by a crystal violet staining method and spectrophotometric measurements, *n* = 9. (**C**) Viability of bacterial cells determined by fluorescent staining with a LIVE/DEAD kit and a ratio of green/red fluorescence, *n* = 3. (**D**) Density of planktonic cells assessed by spectrophotometry, *n* = 9. (**E**) A set of images showing the growth of *H. pylori* 2CML under the most optimal conditions of the current model—BHI + 5% FCS + 1% SGF (marked with red bars). Scale bars, 20 µm. * indicates statistical difference (*p* < 0.05) with the control—0%.

The most well-known type of secretion produced by the stomach is gastric juice (gastric fluid) [40,41]. Its main function is to inactivate swallowed microbes and prevent them from reaching the intestines. Thus, gastric juice is frequently considered as the first line of defense against infections throughout the gastrointestinal tract. Therefore, in the second stage of our model, we decided to assess whether the presence of gastric fluid in the classically used culture medium (BHI + 5% FCS) affects the physiology of *H. pylori*. As in the previous experiments, we assessed the effect of the concentration gradient of SGF on the optical density of planktonic forms, viability (measured with the LIVE/DEAD kit), autoaggregation degree, and the amount of biofilm formed (Figure 3).

In a validated version of the experiments, we narrowed the concentration gradient of SGF to 0–2% (in 0.2% intervals). This decision was dictated by the death of bacteria both during incubation in pure SGF (already after 30 min) and the originally planned concentrations in the range of 0–10%. This situation is most likely related to the fact that *H. pylori*, contrary to popular belief, is not an acidophilic bacterium [42]. Contact with a very low gastric pH in vivo, such as in the SGF applied in our studies (pH = 1.6), is very short. Avoidance of the acidic environment by *H. pylori* is associated with an intensive production of urease and the presence of flagella, which are additionally accompanied by a negative chemotaxis against protons [43]. These features ultimately lead to a rapid and directional localization of *H. pylori* within the gastric mucosa, where the prevailing pH is typically in the range of 6–7.4 [42,44,45]. Thus, in this series of experiments, we set out to determine how small changes in the environmental acidification induced by low concentrations of SGF affect the physiology of this bacterium.

We observed that concentrations between 0.2% and 1% of SGF gradually stimulated two of the four parameters assessed (autoaggregation and biofilm amount), with the effect on the viability often being inversely proportional (specifically well seen during 2- and 3-day culture) (Figure 3). Although 0.2–1% SGF slightly increased the optical density of planktonic cells, this effect was mostly insignificant. Additionally, when a concentration of 1% SGF was exceeded, the autoaggregation- and biofilm-stimulating effect was gradually lost, and the values of the assessed parameters matched those observed for control samples (0% SGF). Firstly, in the current series of experiments we confirmed that a 3-day incubation of *H. pylori* determines the most robust biofilm production. Once again, we associate this fact with the increase in AI-2 concentration together with a prolonged culture and the resulting dispersion of the *H. pylori* biofilm during a one-week incubation. Secondly, during the 2- and 3-day cultures, we noticed a very strong decrease in the green/red fluorescence ratio after LIVE/DEAD staining, especially for the SGF concentrations most favorable for intensifying all other biological parameters, e.g., biofilm formation (Figure 3). Interestingly, this effect was completely eliminated during the 7-day culture, questioning our original assumption that the increase in red fluorescence was the result of bacterial cell death. We suspect that this phenomenon is correlated with the accumulation of extracellular DNA (eDNA) in the biofilm matrix. As a confirmation of our hypothesis, we would like to draw attention to the previous results by our team [16,17] and others [46,47,48], demonstrating the importance of eDNA as a building unit of *H. pylori* biofilm. We believe that accumulated eDNA in the biofilm matrix of *H. pylori* reacts with the propidium iodide of the LIVE/DEAD kit and enhances the red fluorescence signal. This type of mechanism was described in detail several years ago by the team of Rosenberg et al., who showed with various bacterial species that eDNA in the biofilm matrix interferes with the interpretation of LIVE/DEAD staining and, as a result, underestimates the recorded bacterial viability [49]. Thus, based on all the above considerations, we suspect that exposure of *H. pylori* to SGF results in the accumulation of eDNA and related with this stimulation of both autoaggregation and biofilm formation. This effect disappears during longer incubation in the above conditions, most likely as a result of the use of eDNA as a nutrient source [50] or due to the uptake of this genetic material during the transformation process of naturally competent *H. pylori* [51], although these assumptions must be confirmed experimentally in the future.

**Figure 4:**
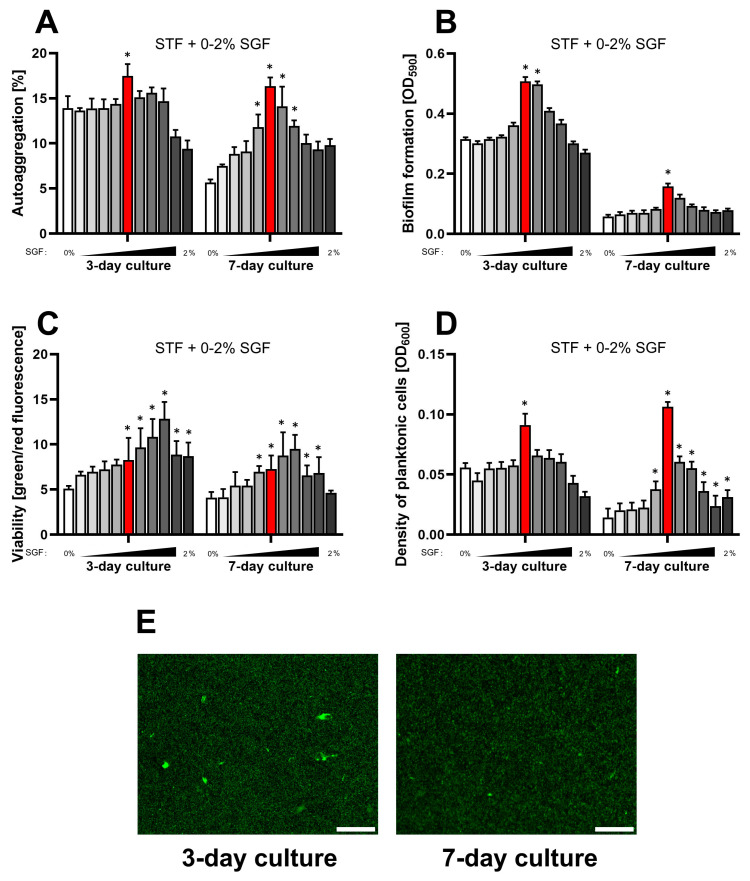
Assessment of the impact of a one-week culture on the physiological parameters of *H. pylori* 2CML in STF and the concentration gradient of SGF (0–2%, with 0.2% intervals). An increasing concentration of SGF is marked by the intensified gray of bars. (**A**) Autoaggregation measured microscopically by estimating the degree of the observation field coverage, *n* = 3. (**B**) Biofilm formation determined by a crystal violet staining method and spectrophotometric measurements, *n* = 9. (**C**) Viability of bacterial cells determined by fluorescent staining with a LIVE/DEAD kit and a ratio of green/red fluorescence, *n* = 3. (**D**) Density of planktonic cells assessed by spectrophotometry, *n* = 9. (**E**) A set of images showing the growth of *H. pylori* 2CML under the most optimal conditions of the current model—STF + 1% SGF (marked with red bars). Scale bars, 20 µm. * indicates statistical difference (*p* < 0.05) with the control—0%. Because of the low values for all the physiological parameters during 1- and 2-day cultures, these data were not recorded.

In the second stage of the current series of experiments, we decided to verify the physiological parameters of *H. pylori* during exposure to low concentrations of SGF when STF was used as a primary culture medium (Figure 4). We hoped that this type of procedure would make it easier to interpret the results, due to the reduction in the amount of culture media components and potential factors that could influence the observations. Indeed, this management confirmed that the use of low concentrations of SGF (max 1%) had a beneficial effect on the biological parameters we assessed. Again, we were able to confirm that a 3-day incubation resulted in a greater amount of biofilm formation of *H. pylori* than a 7-day culture period. Interestingly, unlike the previous culture conditions (BHI + 5% FCS + 0–2% SGF), in the current culture conditions concentrations between 0.2% and 1% SGF did not negatively affect the viability readings. We suspect that in culture media with a very rich chemical composition, the availability of substances allows for the intensive accumulation of biofilm matrix (including eDNA) [52] and therefore may be associated with a greater risk of misinterpreting the viability of biofilm cells. Nevertheless, observations of the current series of experiments confirmed our suspicions that 1% SGF had no negative effect on *H. pylori* viability and that the low green/red fluorescence ratio detected in our previous experiments resulted from eDNA accumulation in the biofilm matrix.

### 2.4. Summary of the Impact of STF and SGF on the H. pylori Physiology

Summarizing the results of a series of detailed experiments aimed at determining the most favorable in vitro conditions stimulating biofilm formation by *H. pylori* in an environment imitating the gastric milieu (Figure 1, Figure 2, Figure 3 and Figure 4), we have shown that the use of STF as a basic culture medium together with 8% FCS and 1% SGF is the most optimal to achieving our research goal and will be applied in the next stages of our experimental analyses. In this context, it is worth mentioning that although artificial media (e.g., BHI) supplemented with simulated human fluids (STF and SGF) can intensify the biofilm formation of *H. pylori* even more, their nutritional richness and the resulting strong stimulation of bacterial proliferation have little in common with the conditions encountered by bacteria in vivo and will therefore be rejected at this stage of research.

### 2.5. Selection of a Gastric Cell Line

To further improve our research model and increase the similarity to conditions encountered by *H. pylori* in the stomach, in the next step we decided to focus on obtaining a co-culture of this bacterium with a gastric cell line. At this point, we were faced with a decision to choose the most appropriate among the many that were commonly available. The most important criterion that guided us when selecting the cell line for the current study was the profile of mucins produced. Numerous literature data show that one of the primary receptors on the gastric mucosa for *H. pylori* is MUC5AC [53], while during infection caused by this bacterium, a strong decrease in the amount of this mucin in favor of an increase in MUC5B and MUC6 is observed [54]. As a result of our preview of data on phenotypic features presented by various gastric cell lines, we decided that KATO III (HTB-103) would be the most suitable for the current model. The cells constituting this line were first obtained in 1978 from a 55-year-old patient with a gastric signet ring cell adenocarcinoma [55]. In the study by Matsuda et al., it was observed that among the tested gastric cell lines, only KATO III showed a decrease in MUC5AC expression as a result of exposure to *H. pylori* in vitro [56]. Additionally, in another study comparing two cell lines, AGS and KATO III, it was noticed that they presented low and high levels of MUC5B expression, respectively [57]. The results presented above highlight similarity in the mucin profile between KATO III cultured in in vitro conditions [56,57] and gastric mucosa reported in vivo [54]. Therefore, in the present research of our team, we decided to choose this cell line.

### 2.6. Establishment of H. pylori Co-Culture with KATO III in a Semi-Permeable Membrane Model

In the first step of this series of experiments, we set out to determine whether the KATO III line is able to grow and form a uniform monolayer when cultivated in conditions resembling the stomach environment. For this purpose, we performed a pilot culture of these cells in STF supplemented with either 10% or 20% of Fetal Bovine Serum (FBS). We noticed that in both cases, the cells of this line multiply; however, faster obtaining of the proper confluence was achieved in the STF + 20% FBS medium. Therefore, in the next experimental stages, we decided to incubate KATO III in these conditions.

Knowing the culture conditions of *H. pylori* and KATO III, in the next stage we attempted to obtain a co-culture of both using a semi-permeable membrane model. According to the literature [58], porous semi-permeable membranes are a very important tool in achieving the proper growth of cell lines. This property is closely connected with an ability to achieve compartmentalization, allowing for the use of different culture media from the apical and basolateral sides. Additionally, apart from optimizing the growth of cell lines, this system also facilitates suitable cell imaging without disturbing delicate cell–cell interactions.

**Figure 5:**
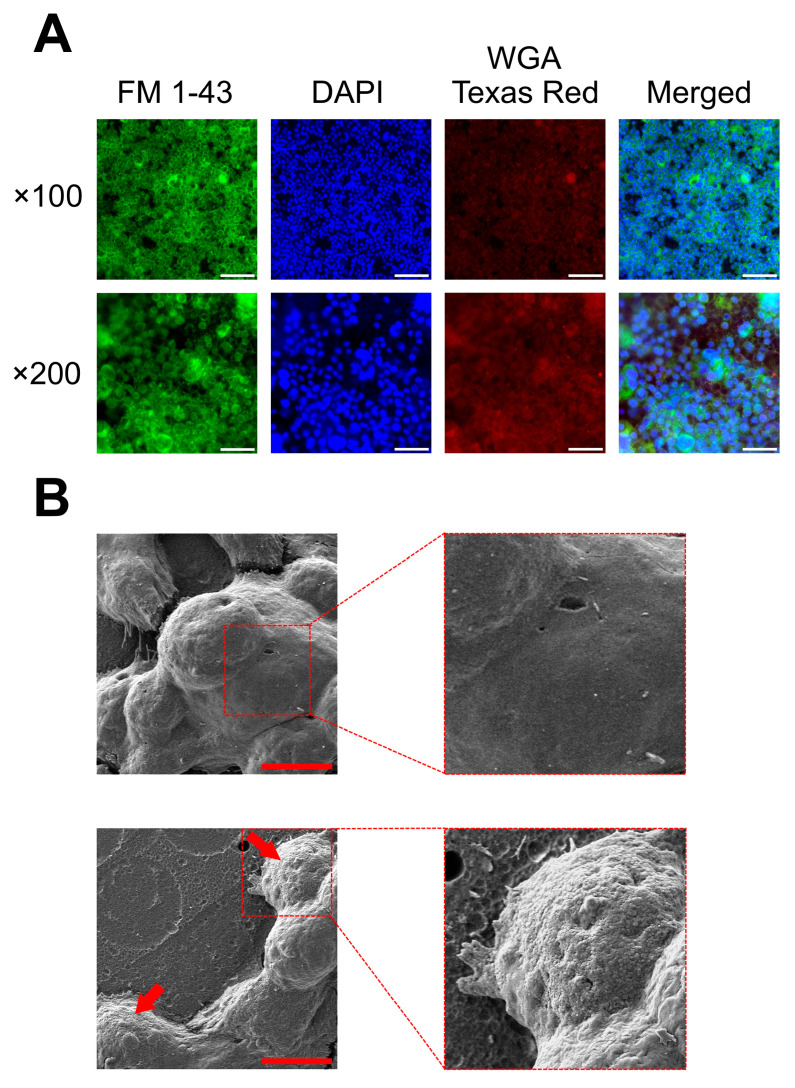
Representative images showing a co-culture between *H. pylori* 2CML and KATO III cells growing on semi-permeable membranes in host-mimicking fluids. (**A**) A set of images obtained by fluorescence microscopy with different components of the co-culture model being stained (FM 1–43 to visualize bacterial biomass (biofilm), DAPI to show cell nuclei of eukaryotic cells, and WGA-conjugated Texas Red to indicate the presence of mucus glycoproteins (mucins)). Scale bars, 20 µm. (**B**) A set of images obtained by scanning electron microscopy. On the top, a control constituting non-infected KATO III cells; on the bottom, a co-infection of KATO III with *H. pylori* 2CML, where red arrows indicate areas of biofilm development. Scale bars, 20 µm.

Keeping in mind the benefits of using the culture on semi-permeable membranes, we applied a two-stage system to obtain a co-culture of bacterial cells with a gastric cell line (Figure 5). In the first stage, we incubated KATO III for 3 days on semi-permeable membranes, where these cells had access to STF + 20% FBS medium from both the apical and basolateral sides. This procedure was aimed at obtaining an optimal growth of KATO III on the semi-permeable membranes. In the second stage, the medium along with the unadhered cells from the apical side was removed and replaced with STF + 8% FCS + 1% SGF medium containing a suspension of *H. pylori*. Such a system was cultured for another 3 days to obtain an appropriate bacteria–gastric cell interaction. The undoubted advantage of using the compartmentalization of culture media in this system was the possibility of creating a gradient of chemical conditions, i.e., more acidic and nutrient-poor from the apical side, while more pH-neutral and rich in nutritional composition from the basolateral side. This type of approach correctly reflects the physicochemical conditions encountered by *H. pylori* in the stomach and forces this bacterium to activate chemotactic reactions towards the gastric cell line (chemoattractant towards the source of nutrients and chemorepellent towards the acidic environment) [43,59].

Fluorescence observations of semi-permeable membranes containing a co-culture of KATO III with *H. pylori* 2CML proved the development of biofilm of this bacterium on the surface of gastric cells (Figure 5). Interestingly, the assessment of co-localization of bacteria with cellular components of KATO III (cell nucleus with a more central localization and mucins with a more peripheral location) did not show significant differences (Figure S1), indicating that after a 3-day co-culture period the localization of *H. pylori* may be more of a random character (Figure S1). Nevertheless, as for now we are not able to clearly reject the possibility that perhaps at earlier stages of co-culture (e.g., lasting several hours), the localization of *H. pylori* on KATO III cells might be more structured. To further confirm the presence of *H. pylori* on the surface of KATO III lines growing on semi-permeable membranes, we performed ultrastructure imaging using SEM (Figure 5). This approach confirmed the presence of bacterial aggregates on the surface of the gastric cell line.

To sum up, using a co-culture system on semi-permeable membranes, we managed to achieve a proper growth of the KATO III monolayer with the *H. pylori* biofilm on its surface in culture media imitating the stomach environment (Figure 5).

### 2.7. Establishment of H. pylori Co-Culture with KATO III in a Microfluidic Model

In the last series of experiments of the current article, we decided to verify the usefulness of a system generating microfluidic conditions as an alternative to culturing *H. pylori* with KATO III in a semi-permeable membrane model. Microfluidics is a technology dealing with systems that analyze biological and/or physicochemical phenomena occurring in small volumes of fluids flowing through microscale channels or chambers [60,61,62]. The advantages of this system include a low consumption of reagents, high throughput, and increased ability to control environmental conditions, all of which in turn positively affect the possibility to analyze cell–cell interactions [60]. One of the pieces of equipment allowing for this type of capability is the Bioflux platform [63]. In the recent research of our team [16], for the first time in the world, this system was successfully used to validate the methodology of *H. pylori* biofilm formation in microfluidic conditions. Therefore, in the current research, we attempted to establish a co-culture of *H. pylori* with KATO III in microfluidic conditions generated by the Bioflux system.

Our pilot attempts to obtain a uniform monolayer of KATO III cells in the microcapillaries of the Bioflux system were unsatisfactory. In this context, different incubation periods (1–3 days) and different adhesion modes (constant-flow or flow-adhesion-flow) were checked. It turned out that despite an initial obtainment of a relatively good degree of microcapillary coverage, consecutive hours contributed to cellular detachment and a collision-dependent removal of subsequent cells located in the more distal fragments of the microcapillaries. The most probable cause of the above phenomenon is the occurrence of three phenotypes of KATO III cells, i.e., adherent, non-adherent, and spheroidal clusters, which can transform into one another [64]. We suspect that in the microfluidic growth model of KATO III cells, the non-adherent and spheroidal cluster phenotypes were responsible for these events.

**Figure 6:**
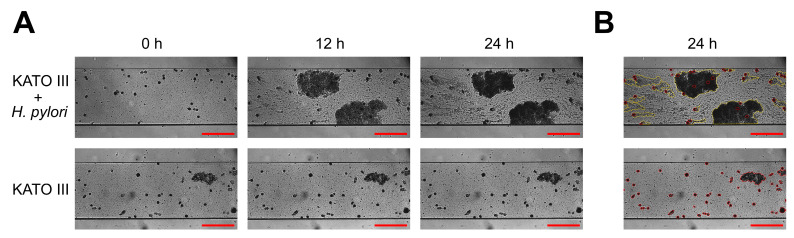
Representative images showing a co-culture between *H. pylori* 2CML and KATO III cells growing in microfluidic conditions in host-mimicking fluids. (**A**) Time-dependent development of *H. pylori* biofilm attached to KATO III cells. (**B**) An image showing a one-day co-culture with a marking of specific components of this model, where red circles indicate KATO III cells and yellow dashed lines highlight areas of the development of *H. pylori* biofilm, being in close contact with these cells. Scale bars, 20 µm. To see stacked time-lapse sequences from the above experiments, please see Videos S1 and S2.

Nevertheless, willing to investigate the usability of microfluidics in assessing the formation of *H. pylori* biofilm on the surface of KATO III cells, we decided to continue the analysis despite the impossibility of obtaining a uniform monolayer of gastric cells in microcapillaries. Time-lapse analysis of the microscopic images of microcapillaries with attached KATO III cells showed the gradational development of *H. pylori* biofilm attached to these gastric cells (Figure 6, and Videos S1 and S2). Especially after a one-day co-culture, it was visible that KATO III cells constituted the primary sites for adhesion of *H. pylori*, which was able to form long tongue-shaped biofilm structures as a result of clonal multiplication and shear flow pressure.

In summary, using a microfluidic culture system, we were able to observe an intimate bacteria–gastric cell interaction during the development of *H. pylori* biofilm in microfluidic conditions and culture media imitating the stomach environment (Figure 6). Despite the above observations, due to the impossibility of obtaining a monolayer of KATO III cells in microfluidic conditions, in future studies applying this type of an experimental model, we propose the use of another gastric cell line with a strong adhesive phenotype.

## 3. Materials and Methods

### 3.1. Storage and Revival of H. pylori

For the establishment of the current research model, a clinical multidrug-resistant *H. pylori* 2CML strain was chosen. This isolate belongs to the collection of the Department of Microbiology of the Wroclaw Medical University and was primarily characterized during our previous studies [16]. The stock culture of the strain was maintained in 30:70 glycerol–Tryptic Soy Broth (TSB; Oxoid, Dardilly, France) at −80 °C, until experiments were carried out. Regrowth of the stain after thawing was performed by spreading the bacteria on Columbia agar plates (Difco, Lublin, Poland) with 10% horse blood (GRASO, Starogard Gdanski, Poland). Such plates were cultured for 3 days at 37 °C in a microaerophilic atmosphere (5% O_2_, 15% CO_2_, 80% N_2_; generated by Genbox microaer kits, BioMerieux, Marcy I’Etoile, France). Then, to obtain the required physiological activity, the strain was inoculated onto fresh agar plates and incubated for another 3 days under identical conditions.

### 3.2. Influence of the Composition of Culture Media on the Physiology of H. pylori

The assessment of the impact of various culture media on the physiological parameters of *H. pylori* was performed using broth cultures in 24-well ventilated titration plates (Bionovo, Legnica, Poland). The primary control medium consisted of BHI (Oxoid, Dardilly, France) with 5% FCS (Gibco, Paisley, Scotland, UK), which constitutes one of the classically used media in the biofilm development of *H. pylori*, as reported by our team [17,65] and others [66,67]. STF (BZ279; BioChemazone, Edmonton, AB, Canada; Figure S2) and SGF (BZ175; BioChemazone; Figure S3) were chosen as host-mimicking fluids. A selection of these two was associated with the fact that the production of these fluids is physiologically connected with the gastric mucosa [32,40,41], a place colonized by *H. pylori* [33,34]. The pH values of BHI + 5% FCS, STF, and SGF were 7.0, 7.4, and 1.6, respectively. Changes in the composition of the tested culture media (see below) influenced their final pH values, which were monitored using a pH meter (Mettler Toledo S.p.A., Milan, Italy). For the precise data regarding the pH values of the examined media, see Figure S4. The tested culture media variants included BHI + 5% FCS with a concentration gradient of STF (0–10%; with 1% intervals), BHI + 5% FCS with a concentration gradient of SGF (0–2%; with 0.2% intervals), STF with a concentration gradient of FCS (0–10%; with 1% intervals), and STF with a concentration gradient of SGF (0–2%; with 0.2% intervals).

In each of the above variants, the wells of titration plates were filled with a final volume of 1 mL of the tested culture medium along with a *H. pylori* suspension with a final density of 10^7^ CFU/mL. The titration plates prepared in this way were subjected to a 3-day incubation at 37 °C, microaerophilic conditions, and shaking at 100 rpm (MaxQ 6000, ThermoFisher, Waltham, MA, USA). After the designated incubation period, four parameters were analyzed each time: optical density of planktonic cells, viability of bacteria, degree of autoaggregation, and biofilm formation.

The optical density of planktonic forms was assessed spectrophotometrically [17,68]. For this purpose, 0.2 mL of a bacterial susception was taken from each well of a 24-well titration plate containing a bacterial culture and transferred to a 96-well microtiter plate (Bionovo, Legnica, Poland). The optical density reading was performed at OD = 600 nm using an Asys UVM 340 microplate reader (Biochrom Ltd., Cambridge, UK). Each time, the values of negative controls (pure culture medium without bacteria) were subtracted from the values of the remaining samples. Measurements were performed in three biological repetitions with three technical replications (*n* = 9).

The viability of bacterial cells and the degree of autoaggregation were assessed using fluorescent staining and microscopic observations [16,17,69]. In this context, 0.1 mL of a bacterial suspension was taken from each well of the 24-well titration plate containing a bacteria culture and transferred to Eppendorf tubes (Bionovo, Legnica, Poland). Bacterial cultures were centrifuged for 10 min at 8000× *g* (Gusto High-Speed Mini Centrifuge; Heathrow Scientific LLC, Vernon Hills, IL, USA). The supernatant from each Eppendorf tube was discarded and the bacterial pellet was suspended in 0.1 mL of a 0.85% NaCl solution. Then, 0.6 µL of a mix of both dyes (1:1 ratio) from the LIVE/DEAD kit (L10316, ThermoFisher, Waltham, MA, USA) was added to each Eppendorf tube. The Eppendorf tubes with bacteria were stored in the dark at room temperature for 15 min to allow for adequate penetration of the dyes. After this time, 20 µL was taken from each Eppendorf tube and spotted on glass slides, which were then subjected to observations using a Carl Zeiss inverted fluorescence microscope (Zeiss GmbH, Jena, Germany). Post-microscopic analysis of the obtained photographs was performed using the ImageJ software version 1.54j. Autoaggregation was interpreted as the degree of bacterial coverage of the observation field, while the bacterial viability was calculated based on the ratio of the intensity of green fluorescence (live cells) to red fluorescence (dead or damaged cells). Measurements of both parameters were made in three independent biological repetitions (*n* = 3).

The amount of biofilm formed was assessed by a crystal violet staining and spectrophotometry [16,17,69]. From each well of a 24-well titration plate containing a bacterial culture, the remaining bacterial suspension was removed. Next, the wells of the titration plate were washed with 1 mL of PBS (Merck, St. Louis, MO, USA), dried, and stained with 1 mL of a 0.1% crystal violet solution (Merck) for 15 min. After this step, the remaining solution of crystal violet was discarded, the wells were washed twice with PBS, dried, and flushed with 1 mL of 96% ethanol (Stanlab, Lublin, Poland). The titration plate was left for another 15 min incubation to dissolve the crystal violet adsorbed in the *H. pylori* biofilm. Then, 0.2 mL of this solution was taken from each well of the 24-well titration plate and transferred to a 96-well microtiter plate. The absorbance of the samples was read at a wavelength of OD = 590 nm using an Asys UVM 340 microplate reader. Each time, the values of negative controls (pure culture medium without bacteria) were subtracted from the values of the remaining samples. Measurements were performed in three biological repetitions with three technical replications (*n* = 9).

### 3.3. Storage and Revival of KATO III

The human gastric cancer cell line KATO III (HTB-103) was obtained from the American Type Culture Collection and stored deep-frozen in the vapor phase of liquid nitrogen. To revitalize the cells, the content of the vial was transferred to Iscove’s Modified Dulbecco’s Medium (IMDM; ATCC, Manassas, VA, USA) supplemented with 20% FBS (Biowest, Nuaillé, France), a 1:9 mix of 100 U/mL penicillin solution with 100 µg/mL streptomycin solution (both from Merck KGaK, Darmstadt, Germany). After revival, the cells were subjected to centrifugation with a speed of 125× *g* for 7 min, and the obtained sediment was gently suspended in a fresh medium and transferred to a culture bottle (Bionovo). Incubation was carried out at 37 °C in a humidified incubator in conditions enriched with 5% CO_2_ (Heracell 150i, ThermoFisher, Waltham, MA, USA), together with changing the medium to a fresh one every 3–4 days until obtaining a monolayer with 70–80% of confluence. For experiments, the cells coming from the 5th and 6th passages were used.

### 3.4. Co-Culture of H. pylori with KATO III in a Semi-Permeable Membrane Model

To obtain a co-culture of *H. pylori* with KATO III in static conditions, a semi-permeable membrane system (Millicell Hanging Cell Culture Insert, PET 0.4 µm, 24-well; Merck) was applied [70]. A 0.4 mL solution of STF + 20% FBS with KATO III cells (2 × 10^6^) was added into the upper chamber, while the lower compartment (constituting the well of a 24-titration plate) was filled with 0.6 mL of the same medium without the cells. After a 3-day incubation at 37 °C and in conditions enriched with 5% CO_2_, the culture medium from the upper chamber was replaced with 0.4 mL of STF + 8% FCS + 1% SGF medium containing a suspension of *H. pylori* (10^7^ CFU/mL). Such a system was incubated for another 3 days in 37 °C and microaerophilic conditions to allow for the development of *H. pylori* biofilm. After this period, the medium from the upper chamber of the inserts was discarded and the semi-permeable membranes were directed for two types of microscopic examinations, i.e., fluorescent and scanning electron microscopy (SEM).

For fluorescent microscopy, the semi-permeable membranes coming from the inserts were cut out with a scalpel and placed on glass slides. Then, they were stained with 20 µL of a saline solution with a mixture of fluorescent dyes: FM 1–43 (green fluorescence; 0.5% *v/v*), DAPI (blue fluorescence; 1% *v/v*), and WGA-conjugated Texas Red (red fluorescence; 1% *v/v*) (all from ThermoFisher, Waltham, MA, USA) to visualize the biofilm [71], eukaryotic nuclei [72], and mucus glycoproteins (mucins) [73,74], respectively. Observations were performed using a Carl Zeiss inverted fluorescence microscope. Using the Bioflux Montage software version 7.10.3.390 (Fluxion, San Francisco, CA, USA), the degrees of bacteria/eukaryotic nuclei and bacteria/mucins co-localizations were counted. Measurements were performed in three biological repetitions with three technical replications (*n* = 9).

For SEM, the methodology of sample preparation and observation was similar to the previous one of our team [16,17]. The whole inserts were flooded gently with 1 mL of a 2.5% solution of glutaraldehyde (Merck) and incubated for one day at 4 °C. After fixation, the inserts were washed three times in 0.1 M cacodylate buffer (Merck) and passed through an increasing ethanol concentration gradient (30%, 50%, 70%, 90%, and 99.8%). After this procedure, the semi-permeable membranes were cut out with a scalpel. The obtained samples were sputtered with a carbon layer (EM ACE600, Leica Microsystems, Wetzlar, Germany) and observed with a Scanning Electron Microscope Auriga 60 (Oberkochen, Germany).

### 3.5. Co-Culture of H. pylori with KATO III in a Microfluidic System

To obtain a co-culture of *H. pylori* with KATO III in microfluidic conditions, the Bioflux 1000Z (Fluxion, San Francisco, CA, USA) was applied [16,75]. This equipment was additionally combined with a Carl Zeiss inverted fluorescence microscope and a Pecton environmental chamber XL S1 (Carl Zeiss, Jena, Germany), allowing for real-time observations and maintenance of the desired temperature and gaseous conditions, respectively. All experiments were performed at 37 °C and microaerophilic atmosphere.

Several attempts were made to establish a proper growth of KATO III cells in the microchannels in microfluidic culture conditions (different incubation periods of the initial adhesion and different flow modes). A validated procedure allowing for the most suitable growth is as follows.

Initially, all microchannels of 48-well microfluidic plates (Fluxion, San Francisco, CA, USA) were unblocked with a strong stream of STF + 20% FBS (10 dyne/cm^2^ for 10 s). After this, both the input and output wells were emptied, and the input wells were re-filled with 20 µL of 100 µg/mL fibronectin solution (Merck, Taufkirchen, Germany). The flow of the medium was directed toward the output wells with a speed rate of 1 dyne/cm^2^ for 1 min. Next, the microfluidic plate was left for 60 min to permit the microchannels to absorb fibronectin. After this step, the microchannels were washed at a speed of 10 dyne/cm^2^ for 10 s with 0.1 mL of STF + 20% FBS to clear away any unbonded fibronectin. The output wells were emptied and re-filled with 0.1 mL of STF + 20% FBS containing KATO III cells (10^7^ cells/mL), and an output-to-input medium flow of 5 dyne/cm^2^ was switched on for 5 s. Then, the flow was stopped, and the cells were left for a 4 h incubation to allow for their adhesion to the microchannels. Thereafter, 1 mL of STF + 20% FBS was added to each input well and the input-to-output medium flow with a speed of 0.1 dyne/cm^2^ was activated for 24 h. Ater a one-day incubation, all the wells were emptied, and the input wells were re-filled with 1 mL of STF + 8% FCS + 1% SGF medium containing a suspension of *H. pylori* (10^7^ CFU/mL). In the control samples, a pure culture medium without bacteria was added. Next, the input-to-output medium flow at a speed of 0.1 dyne/cm^2^ was activated for another 24 h. During this process, photographs of the microchannels were automatically taken at each hour. The experiments were performed in two biological repetitions with three technical replications (constituting images from different microchannels’ fragments) (*n* = 6).

### 3.6. Statistical Analysis

The statistical package R version 4.1.2 was used for data analysis. The data distribution in each group was tested using the Shapiro–Wilk test. The statistical significance of data within and between groups was first determined by the non-parametric Kruskal–Wallis test with the Holm correction for multiple comparison using the package “agricolae” for “kruskal” function. To perform the post-hoc analysis, Dunn’s test with Bonferroni correction was applied. For all tests, RStudio and a significance level of α = 0.05 were used.

## 4. Conclusions

In the current research, we proved the usability of host-mimicking fluids both as primary culture media or as stimulators of autoaggregation and biofilm formation of *H. pylori*. We believe that the data presented in this original article may encourage scientists to intensify research on *H. pylori* biofilm in in vitro conditions simulating the gastric environment. These conditions are more similar to those prevailing in the human body and, thus, the results obtained will be more relevant to the real-life scenario. We trust that our model developed herein is highly applicable and can be used in many research sectors that focus on these bacteria, including deepening the knowledge on the physiological behaviors of *H. pylori* or *H. pylori*–gastric cell interactions as well as in the search for more optimal anti-*H. pylori* therapies.

## Data Availability

Data are contained within the article.

## Supplementary Materials

The supporting information can be downloaded at https://www.mdpi.com/article/10.3390/ijms25189839/s1.
