# Supplementary material for: Optimization of Helicobacter pylori Biofilm Formation in In Vitro Conditions Mimicking Stomach
Source: Int J Mol Sci. 2024 Sep 11;25(18):9839. doi: 10.3390/ijms25189839 (PMC11432336; doi:10.3390/ijms25189839)
Supplement: Supplementary file 1 [file ijms-25-09839-s001.zip › Supplemetnary caption.pdf]

**Figure S1**: Analysis of co-localization of *H. pylori* 2CML with KATO III cell components during fluorescence microscopy observation;

**Figure S2:** Certificate of STF presenting information about chemical composition of the product;

**Figure S3:** Certificate of SGF presenting information about chemical composition of the product;

**Figure S4:** A set of data presenting pH values of tested culture media;

**Video S1**: Time-dependent development of biofilm of *H. pylori* 2CML attached to KATO III cells grown in microfluidic conditions in host-mimicking fluids;

**Video S2**: Time-dependent behavior of non-infected KATO III cells grown in microfluidic conditions in host-mimicking fluids.
