# Supplementary figures and images for: Optimization of Helicobacter pylori Biofilm Formation in In Vitro Conditions Mimicking Stomach
Source: Int J Mol Sci. 2024 Sep 11;25(18):9839. doi: 10.3390/ijms25189839 (PMC11432336; doi:10.3390/ijms25189839)

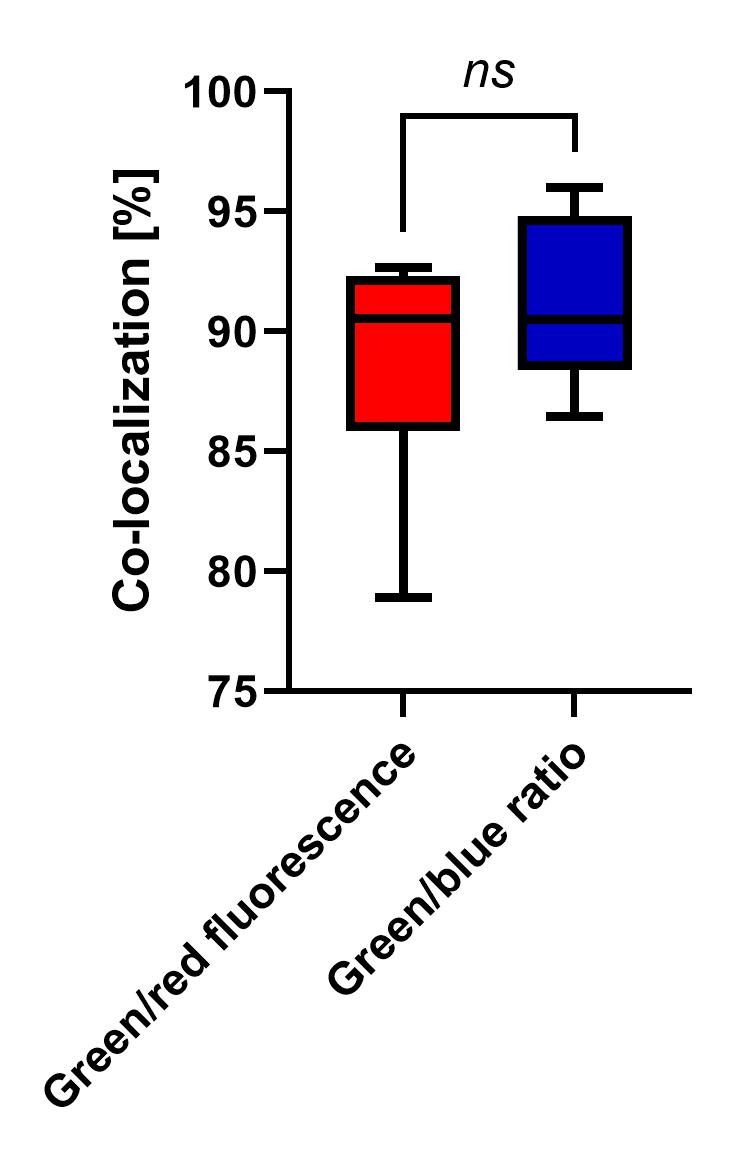

Supplement: Supplementary file 1 [file ijms-25-09839-s001.zip › Figure S1.jpg]

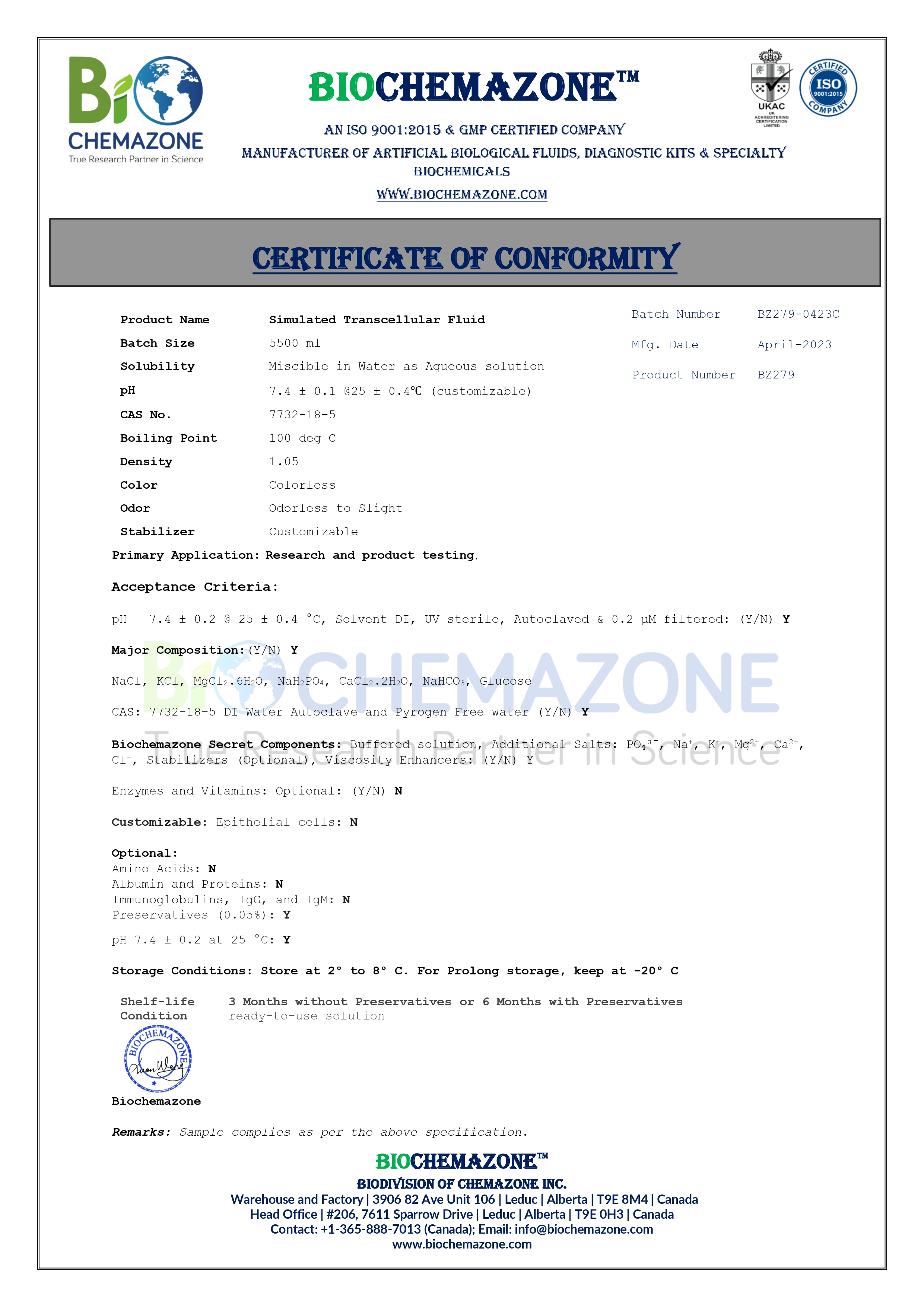

Supplement: Supplementary file 1 [file ijms-25-09839-s001.zip › Figure S2.jpg]

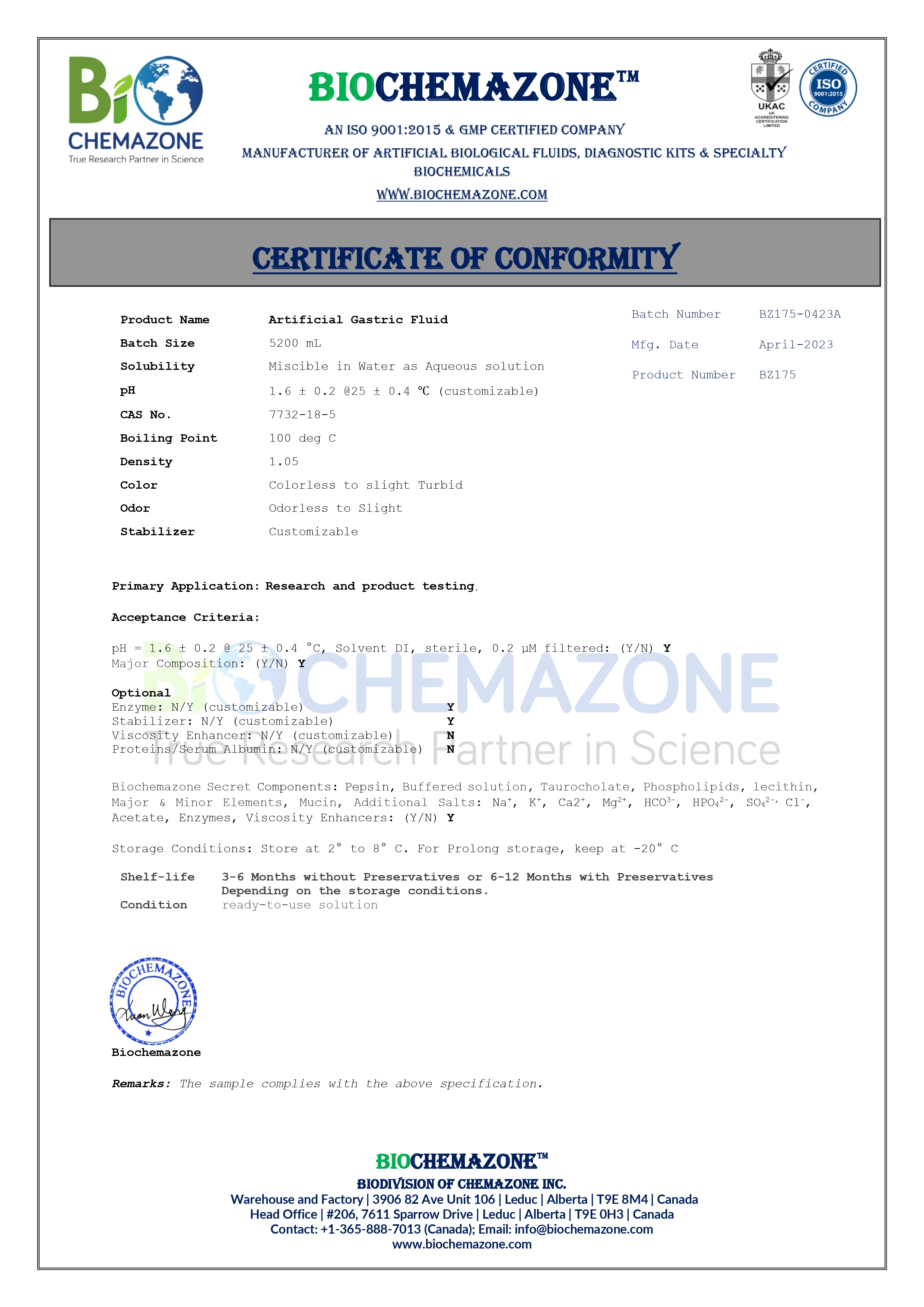

Supplement: Supplementary file 1 [file ijms-25-09839-s001.zip › Figure S3.jpg]

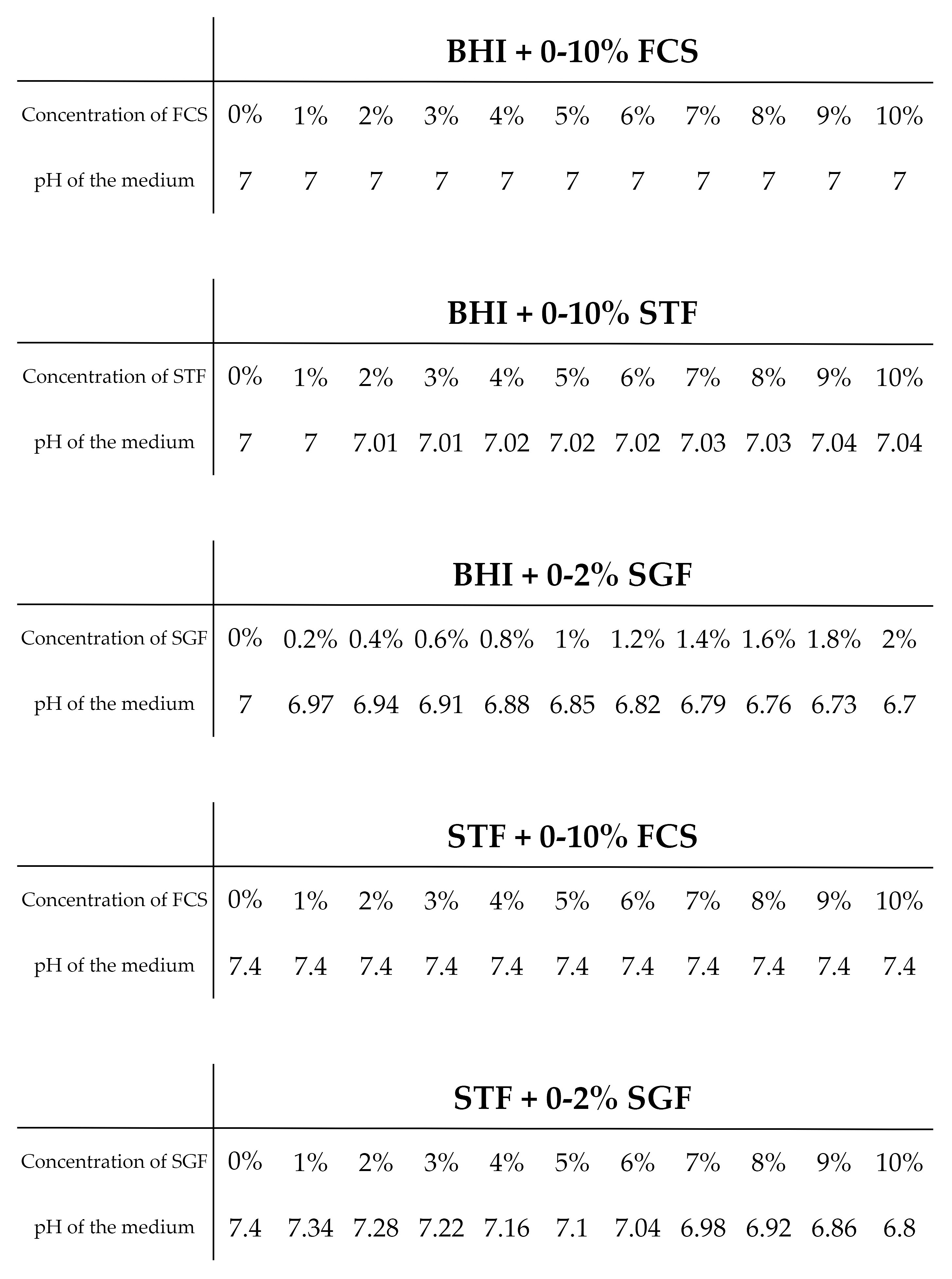

Supplement: Supplementary file 1 [file ijms-25-09839-s001.zip › Figure S4.jpg]
